# Supplementary figures and images for: Strategies for improved endothelial cell adhesion in microphysiological vascular model systems
Source: PLoS One. 2025 May 19;20(5):e0323080. doi: 10.1371/journal.pone.0323080 (PMC12088046; doi:10.1371/journal.pone.0323080)

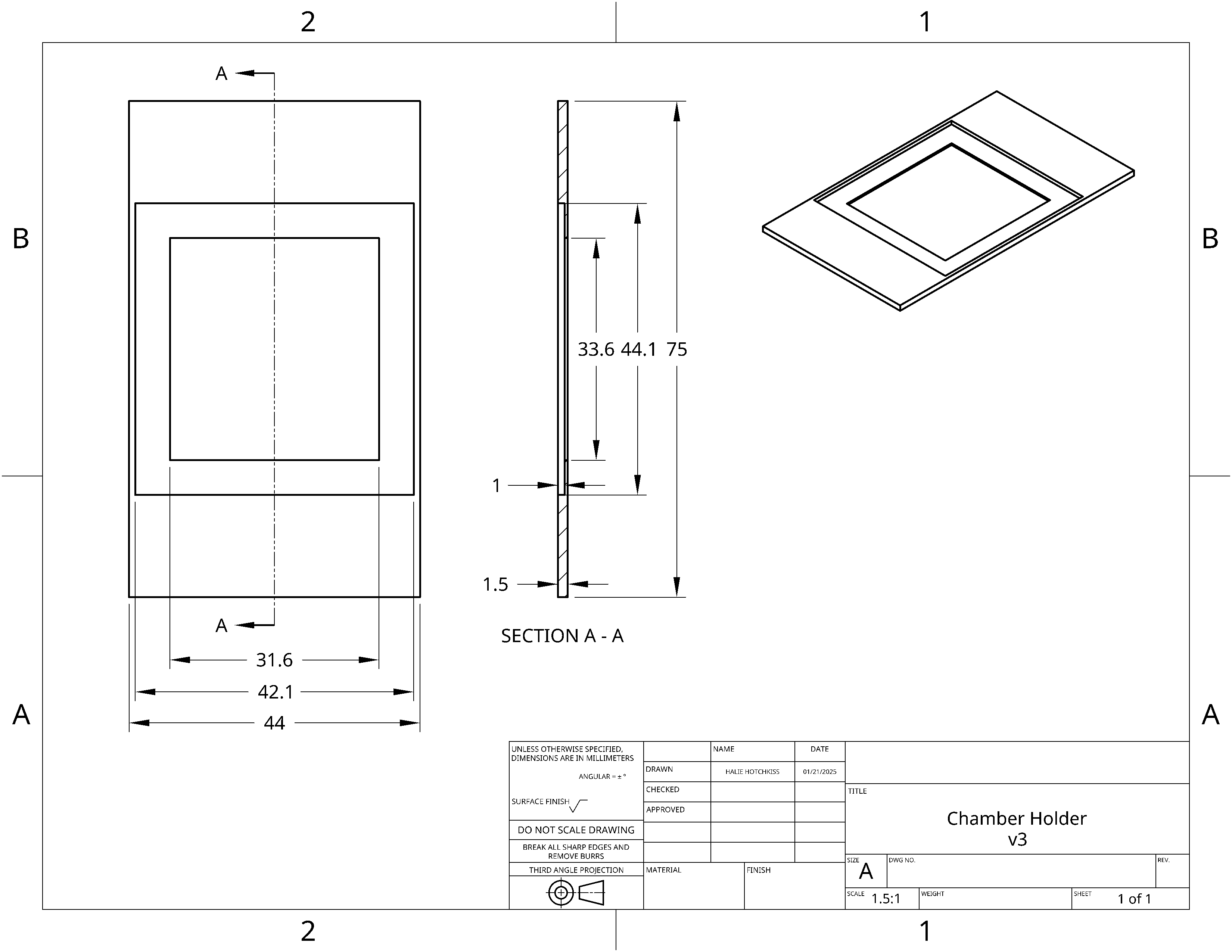

Supplement: S1 File — (ZIP) [file pone.0323080.s001.zip › S1 Chamber Drawings/Chamber Holder v3 Drawing.tiff]

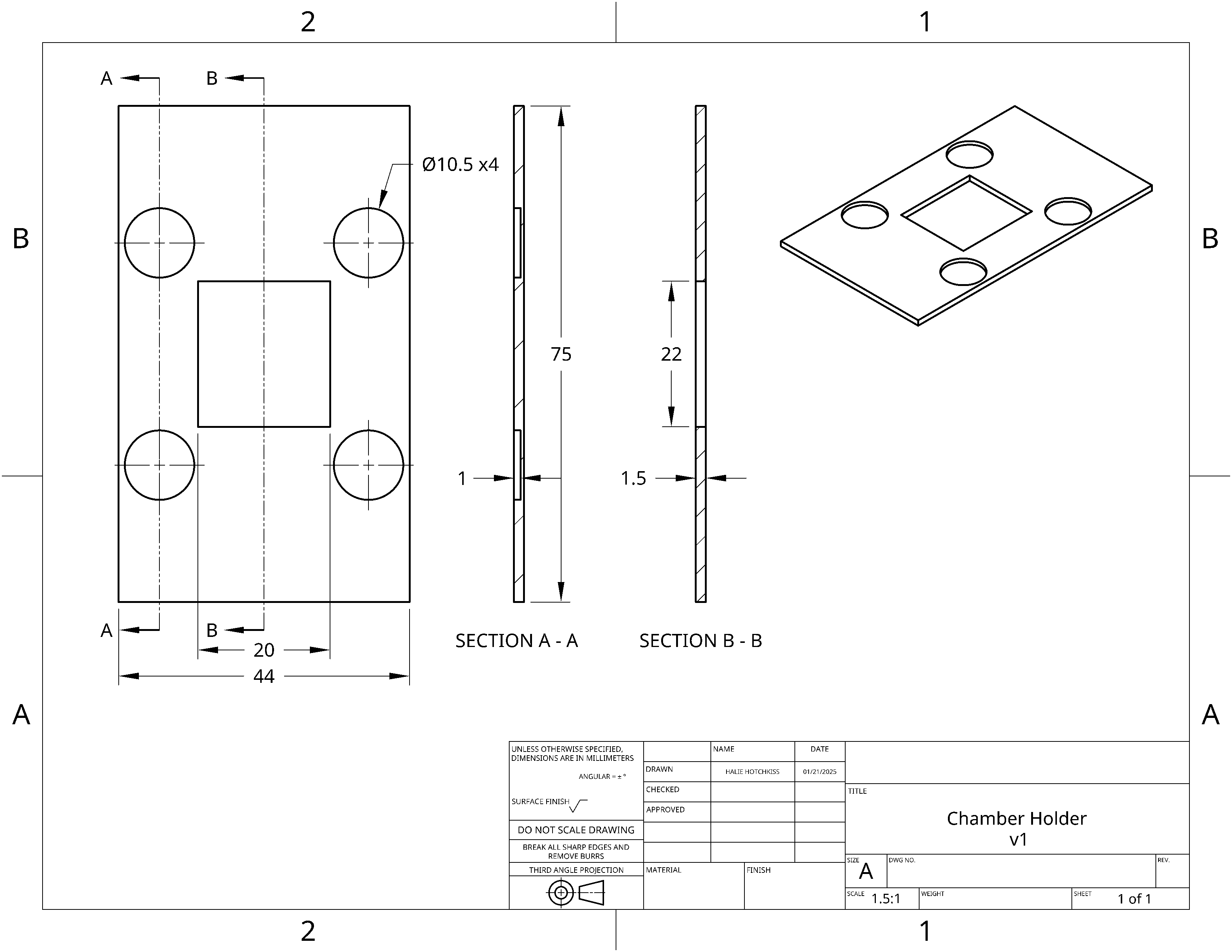

Supplement: S1 File — (ZIP) [file pone.0323080.s001.zip › S1 Chamber Drawings/Chamber Holder v1 Drawing.tiff]

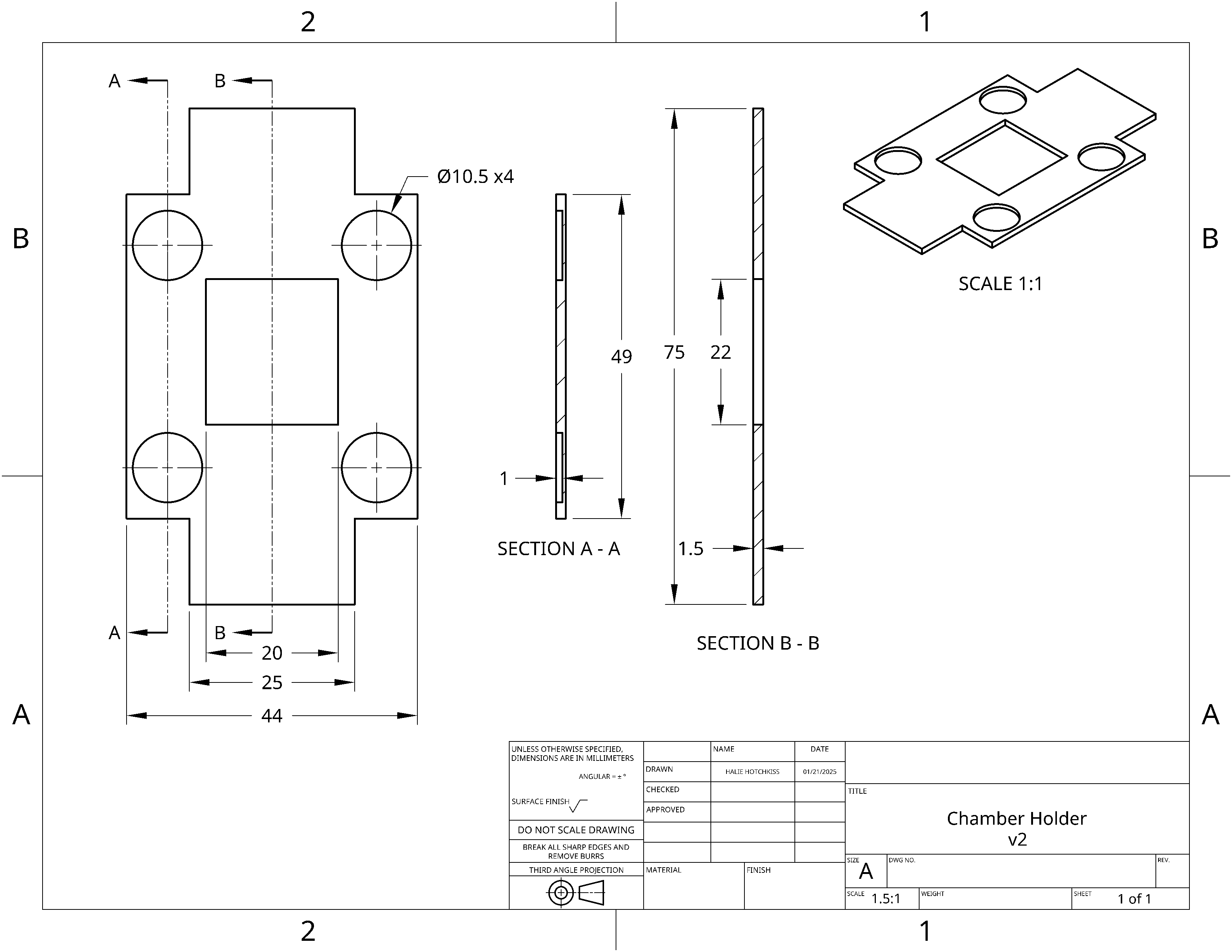

Supplement: S1 File — (ZIP) [file pone.0323080.s001.zip › S1 Chamber Drawings/Chamber Holder v2 Drawing.tiff]

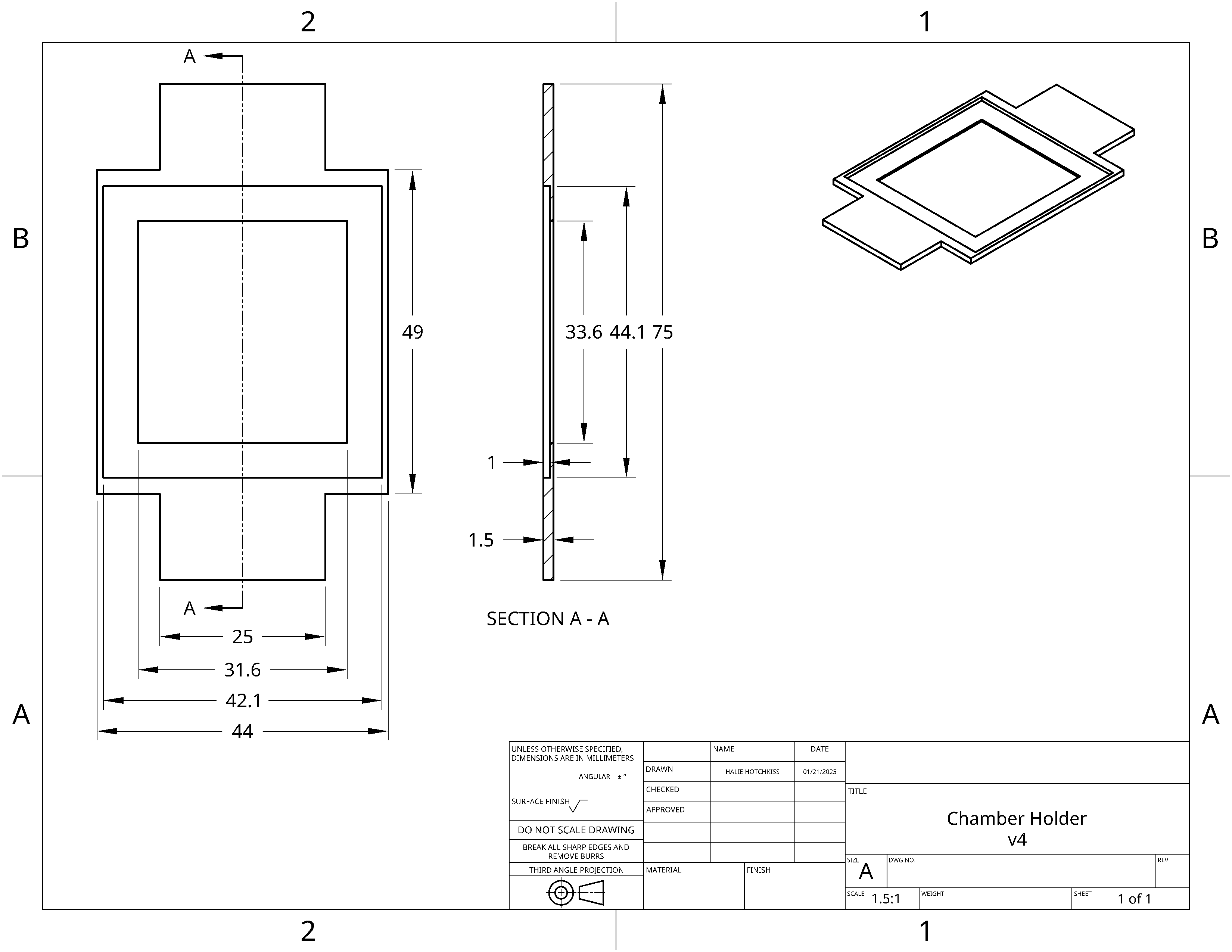

Supplement: S1 File — (ZIP) [file pone.0323080.s001.zip › S1 Chamber Drawings/Chamber Holder v4 Drawing.tiff]

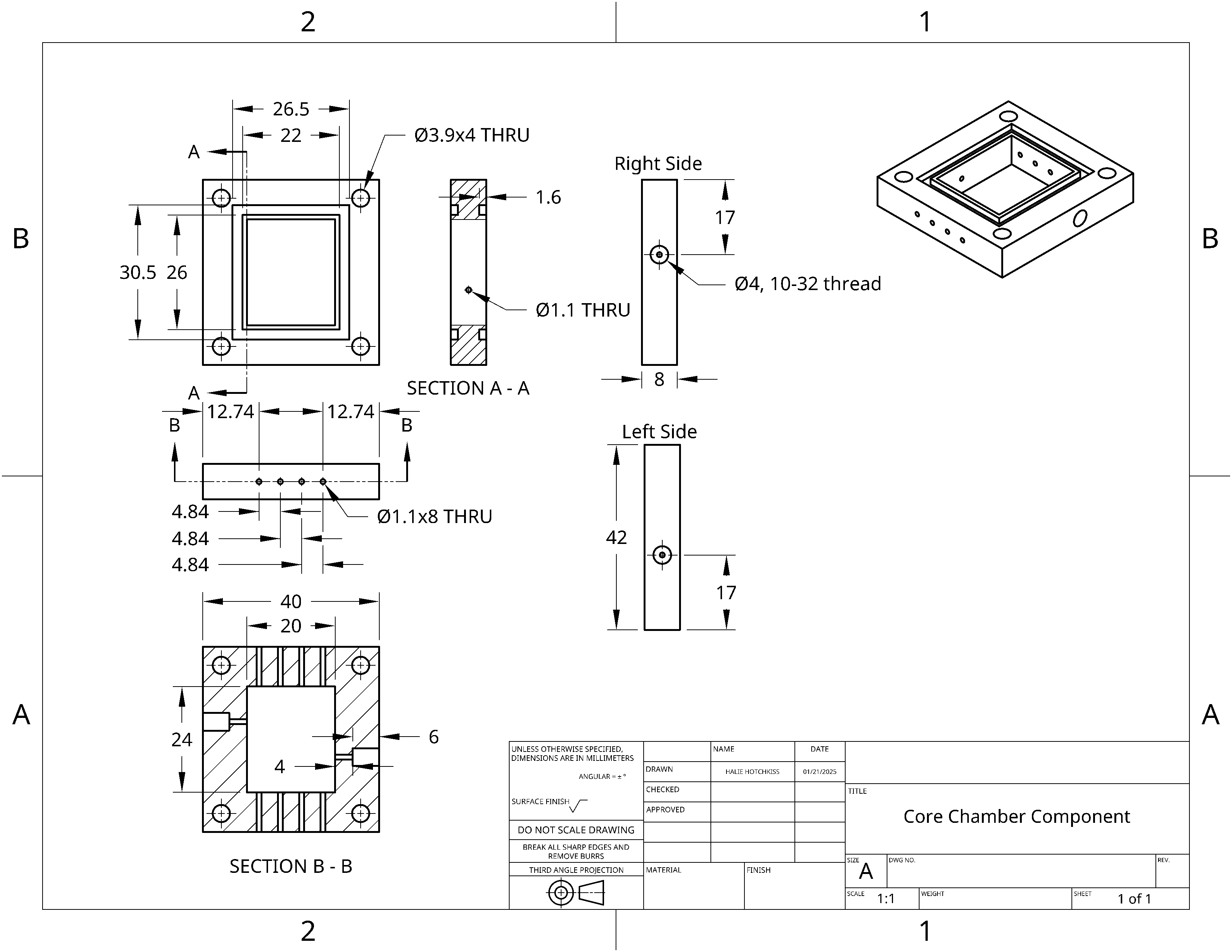

Supplement: S1 File — (ZIP) [file pone.0323080.s001.zip › S1 Chamber Drawings/Core Chamber Component.tiff]

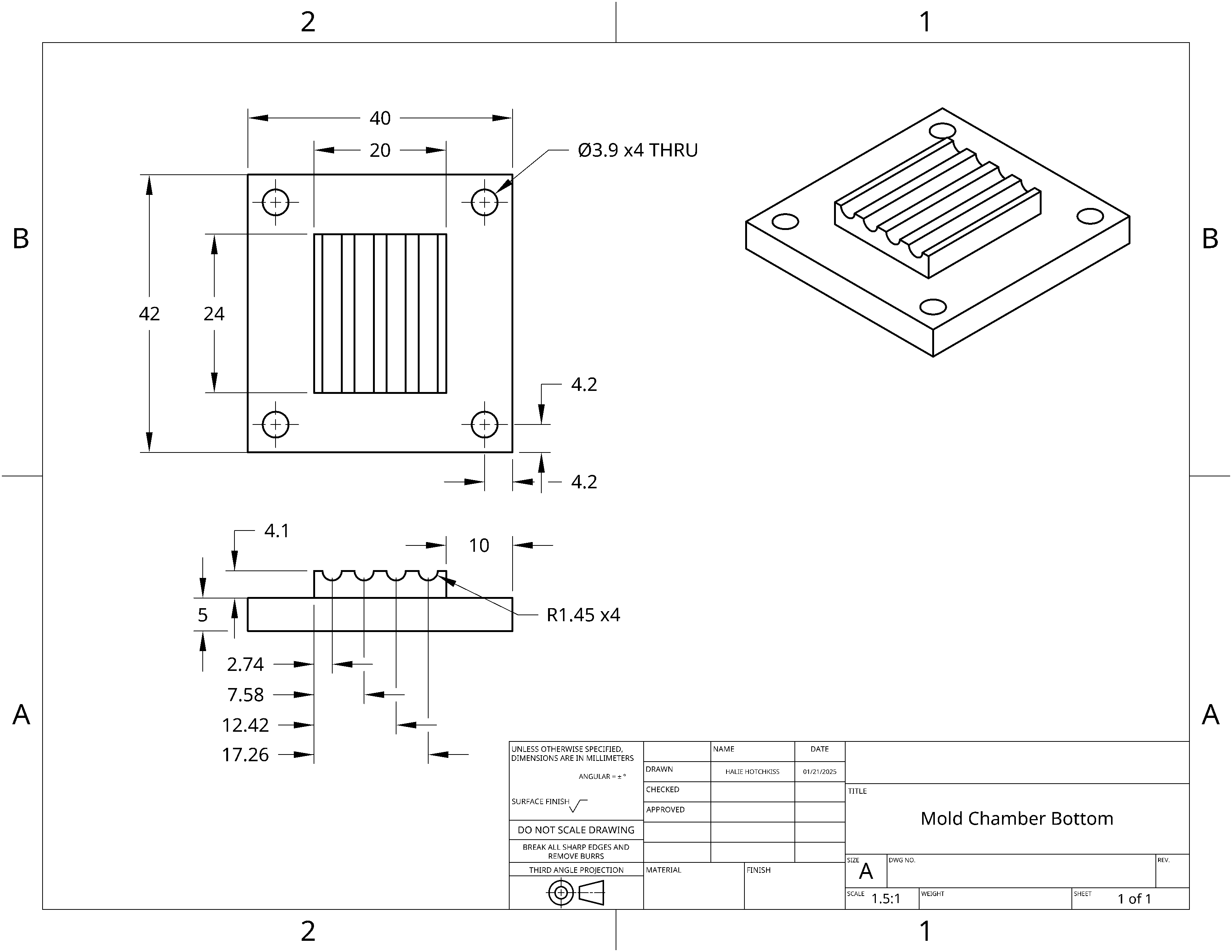

Supplement: S1 File — (ZIP) [file pone.0323080.s001.zip › S1 Chamber Drawings/Mold Chamber Bottom.tiff]

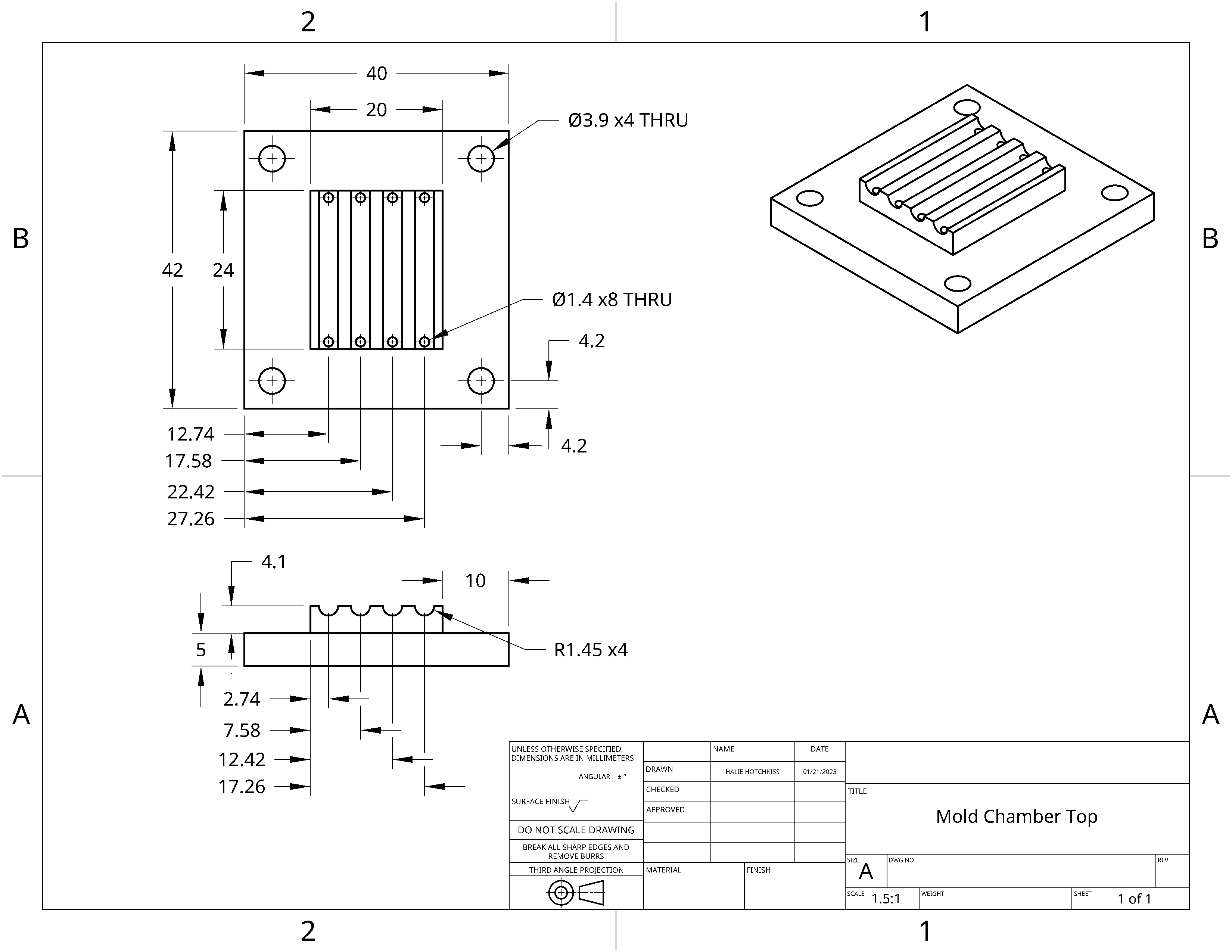

Supplement: S1 File — (ZIP) [file pone.0323080.s001.zip › S1 Chamber Drawings/Mold Chamber Top.tiff]

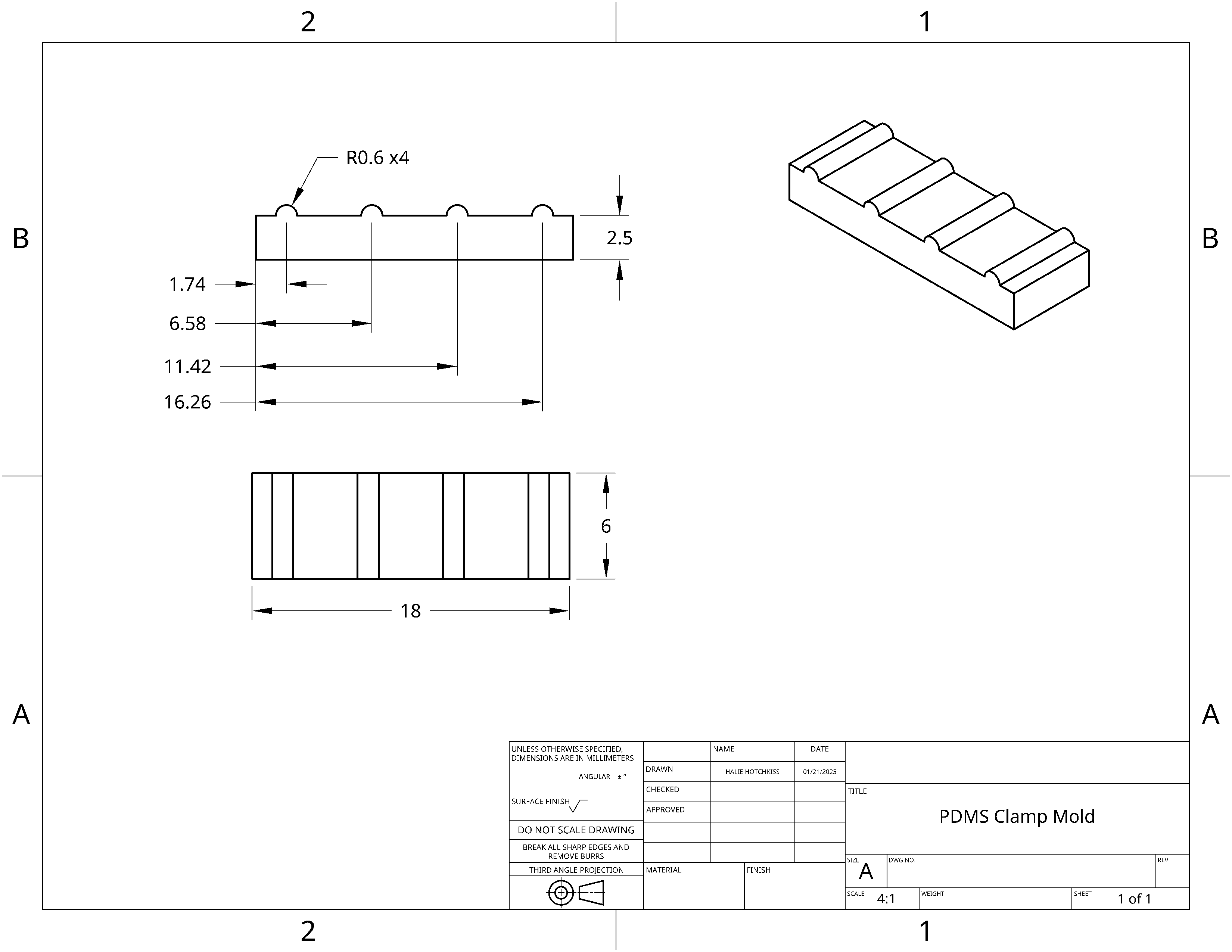

Supplement: S1 File — (ZIP) [file pone.0323080.s001.zip › S1 Chamber Drawings/PDMS Clamp Mold Drawing.tiff]

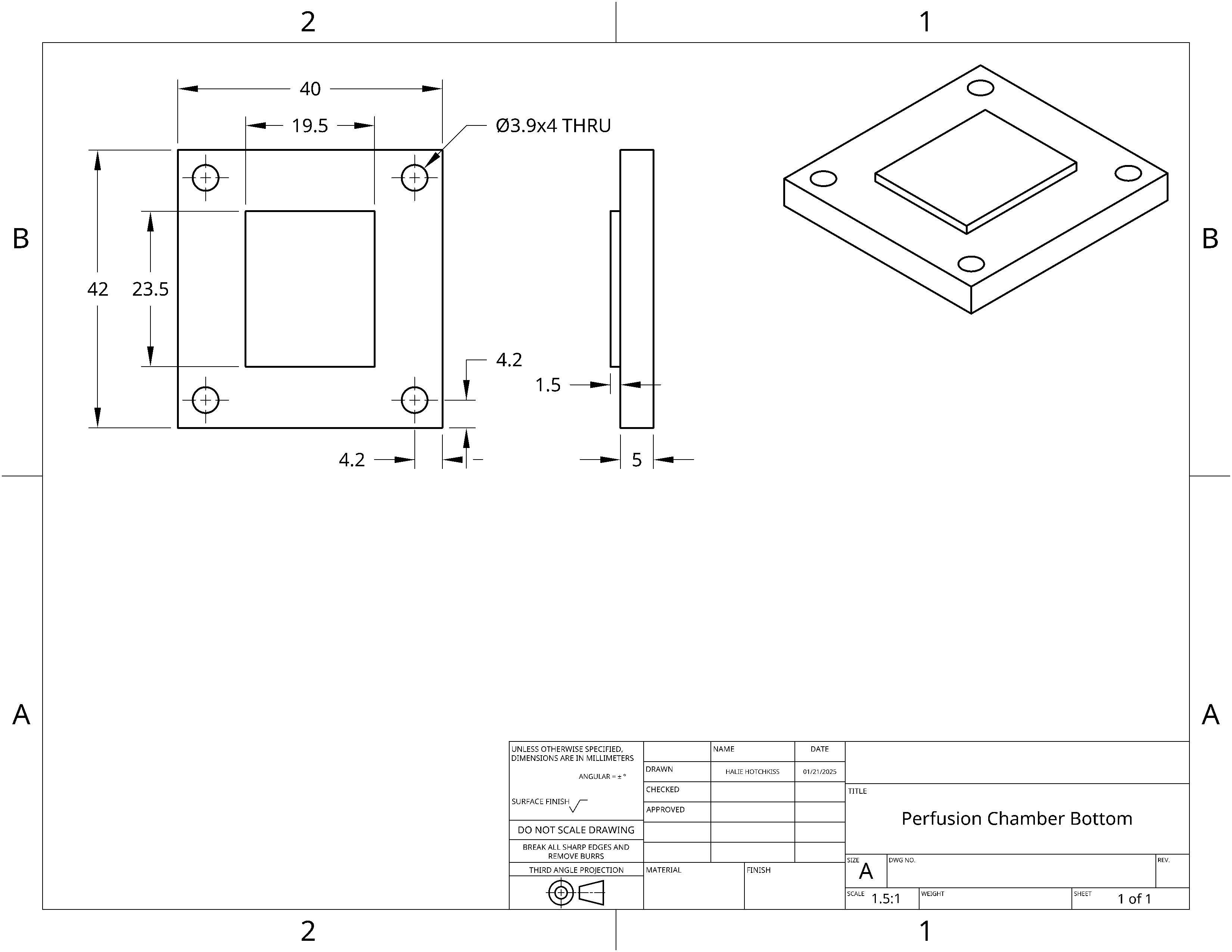

Supplement: S1 File — (ZIP) [file pone.0323080.s001.zip › S1 Chamber Drawings/Perfusion Chamber Bottom.tiff]

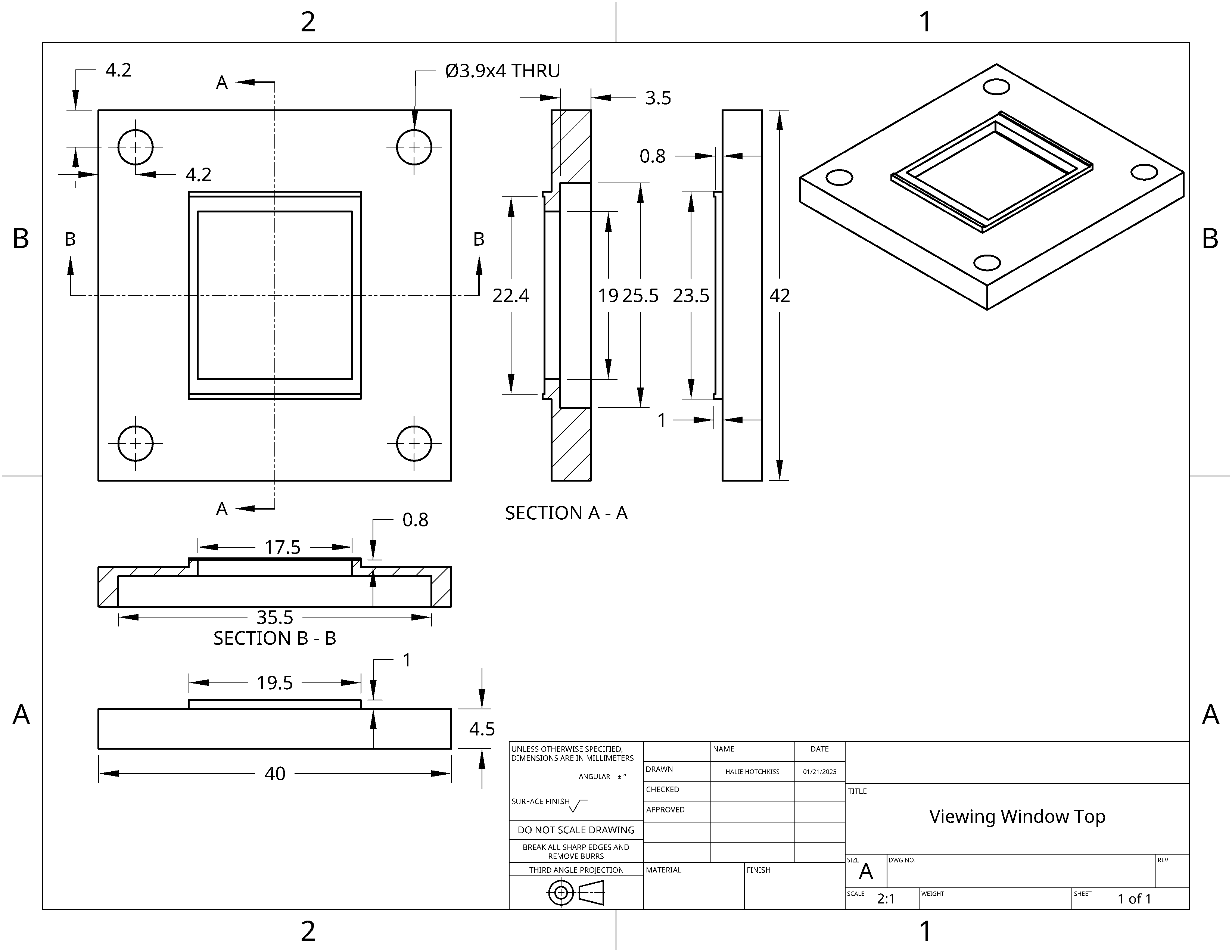

Supplement: S1 File — (ZIP) [file pone.0323080.s001.zip › S1 Chamber Drawings/Viewing Chamber Top.tiff]
