# Supplementary material for: Strategies for improved endothelial cell adhesion in microphysiological vascular model systems
Source: PLoS One. 2025 May 19;20(5):e0323080. doi: 10.1371/journal.pone.0323080 (PMC12088046; doi:10.1371/journal.pone.0323080)
Supplement: S2 File — Also available on protocols.io. (DOCX) [file pone.0323080.s002.docx]

**Supporting File 2. Protocol**

**Strategies for improved endothelial cell adhesion in microphysiological vascular model systems**

Jingyi Zhu^1^, Halie L. Hotchkiss^2^, Kevin L. Shores^1^, George A. Truskey^1*^, Stacey A. Maskarinec^2^

^1^Department of Biomedical Engineering, Duke University, Durham, North Carolina, USA

^2^Division of Infectious Diseases, Duke University Health System, Durham, North Carolina, USA

**Materials**

**Cells**

- Primary human neonatal dermal fibroblasts (hNDFs, Invitrogen, #C0045C), passages 5–11
- Red fluorescent protein (RFP) expressing human umbilical vein endothelial cells (RFP-HUVEC, Angio-Proteomie, #cAP-0001RFP), passages 2-11
- Human umbilical vein endothelial cells (HUVEC, Lonza, #C2517A), passages 2-11
- THP-1 monocytes (ATCC, TIB-202), passages 2-25

Alternative cell sources may be used for fabrication (see reference 4 in the main text).

**Cells culture supplies**

- hNDF growth media, Dulbecco’s Modified Eagle’s Medium (DMEM, Gibco, #11960044) with 4.5 g/L D-glucose supplemented with 10% heat-inactivated fetal bovine serum (Gibco, #10082147), 1% Penicillin/Streptomycin (Gibco, # 15140122), 1 × Non-essential amino acids (NEAA, Gibco, #11140050), 1× Sodium Pyruvate (Gibco, #11360070), 1 × Glutamax (Gibco, #35050061) and 0.1% β -mercaptoethanol (Gibco, #21985023)
- HUVEC and RFP-HUVEC media, Endothelial Cell Growth Medium (Cell Applications, #211-500) supplemented with 1% Penicillin/Streptomycin (Gibco, #15140122)
- THP-1 cell media, RPMI 1640 medium (ATCC modification, Gibco, #A1049101) supplemented with 10% heat-inactivated fetal bovine serum (Gibco, #10082147), 1% Penicillin/Streptomycin (Gibco, # 15140122)
- T75 flasks (Thermo Fisher, #156499)
- 1X Phosphate-buffered saline (PBS, Gibco, #10010023)
- 0.25% Trypsin (Gibco, #25200056), diluted 1:5 in sterile PBS without Ca++ and Mg++
- Gelatin solution (Sigma, #G1393)

**Materials for Polydimethylsiloxane (PDMS) clamps fabrication and sterilization**

- SYLGARD 184 silicone elastomer (PDMS, Dow, #2646340)
- Sterilization Pouch (Cardinal Health, #92510)
- Digital lab oven (VWR # 97025-630)
- Desiccator (Sigma, #Z119008)
- Blade (VWR, # 55411-050)

**Materials for TEBV mold chamber and perfusion/chamber**

- Six computer numerical control machined (CNC) polycarbonate chamber pieces (Protolabs) and one CNC machined viewing top (Protolabs) for viewing chamber (Fig 1)
- Eight stainless steel hypodermic tubes of 0.8” length (20XTW - New England Small Tube Corporation)
- Four stainless steel hypodermic tubes of 3” length referred to as the TEBV mandrels, will be used in the following sections (23RW - New England Small Tube Corporation)
- 22 mm × 40 mm #1.5 Coverglass (VWR, #48393-172), cut into 22mm × 19mm to fit the viewing chamber
- 70A Durometer Buna-N O-Rings, 2 mm wide and 28.5 mm inner diameter (McMasterCarr, #9262K679)
- 1/16” Tube Adapter with 10-32 Threaded Pipe Fitting (McMasterCarr, #2974K123)
- Four pedicle screws with four nuts (6/32 Thread Size 1" long, McMasterCarr, #99607A128 and #91240A007)
- 2 mm biopsy punch (Fisher Scientific, #12460399)
- Teflon tape (Sigma-Aldrich, # 20808-U)
- Epoxy (Henkel, #235033)

**Materials for preparing collagen matrix and harvesting hNDFs**

- Rat-tail collagen I, high concentration (8–10 mg/mL), 100 mg (Corning, #354249)
- Dulbecco’s Modified Eagle’s Medium, Low Glucose, 10X (Sigma, #D2429)
- 1 M NaOH, sterile, prepared by dissolving NaOH pellets in deionized water and sterile filtering after cooling (Sigma, #S8045)
- 2.5 mL Eppendorf tubes (Fisher Scientific, #05-408-138)
- Benchtop centrifuge (Corning, # 6770)

**Materials for TEBV plastic compression/dehydration and endothelialization**

- Two 50 mL Falcon tubes (Corning, #430828)
- One 1 mL sterile disposable syringe (BD Biosciences, #309659)
- One 5 mL sterile disposable syringe (BD Biosciences, #309646)
- 5–6 Kimwipes
- 2 cm × 2 cm Kimwipes (cut)
- One 5 mL syringe adapter (McMasterCarr, #51525K281)
- One 1mL syringe adapter (Strategic Applications Inc., #B23-50)
- L/S® Precision Pump Tubing (as syringe tubing, VWR, # MFLX96410-13)
- One stainless steel forceps, sharp tip, autoclaved (Fisher Scientific, #16-100-113)
- Deep Petri dish (Fisher Scientific, FB0875711)
- Customized rotator with 24 rotations per hour (LABQUAKE SHAKER, Barnstead)
- Mechanical Accurate Countdown Timer with 15 Minute Increments (BN-LINK)

**Materials for TEBV Perfusion**

- Stainless steel tray (SouthPointe Surgical Supply, #RT-1350S)
- Y-shaped connecters, for 1/1600 tube (McMasterCarr, #5117K65)
- Tygon tubing (VWR, #MFLX06419-03)
- L/S® Precision Pump Tubing (VWR, #MFLX96410-13)
- Ismatec 3-stop Microbore Tubing, 0.89 mm ID (VWR, #MFLX95714-26)
- Two-way straight connectors (McMaster-Carr, #5117K41)
- Masterflex L/S Precision Modular Drive pump (VWR, #MFLX07557-10) with a multi-channel pump head (VWR, #MFLX07623-10)
- 0.22 μm syringe filter (VWR, #76479-044)
- 25 mL media reservoir bottle (Corning, #1395-25)
- Glass glue (Loctite, #233841)

**Materials for evaluating monocyte adhesion in TEBVs and** **immunofluorescence staining**

- Human monocytic THP-1 cells, passages 5-20
- 1X Phosphate-buffered saline (PBS, Gibco, #10010023)
- 16% Paraformaldehyde (PFA, Electron Microscopy Sciences, #15710), diluted 1:4 in PBS without Ca++ and Mg++
- Goat serum (Sigma, #G9023)
- 10% BSA: 5 g BSA (Sigma, #A9647) in 50 mL PBS
- 0.1% Triton-X solution: 10 uL Triton X-100 (Sigma, #X100) in 10 mL PBS
- Tween 20 (Bio-Rad, #1706531)
- 30% sucrose solution: 30 grams of sucrose (Sigma, #S0389) in 100 mL PBS
- 5 mL centrifuge tube (Genesee, #24-285)
- ImmEdge Hydrophobic Barrier PAP Pen (Vector Laboratories, #H-4000)
- ICAM-1 antibody (Cell Signaling Technology, #62133S)
- vWF antibody (Sigma, #F3520)
- VE-cadherin antibody (Cell signaling, #2500S)
- Hoechst 33342 (Thermo Fisher Scientific, #H3570)
- CellTracker CMTPX (Thermo Fisher Scientific, #C34552)
- Microscope slides (Globe scientific Inc., #1358W)
- Coverslips (18×18mm, VWR, #48368-040)
- Fine tip scissors (Fine Science Tools, #14084-08)
- Tissue-Tek® O.C.T. Compound (Sakura Finetech, #4583)
- Peel away disposable embedding mold (Electron Microscopy Sciences, #70181)
- Leica SP5 inverted confocal microscope
- Zeiss Axio Imager fluorescence microscope (5X, 10X objectives) outfitted with an AxioCam MRm digital camera with ZEN Pro software


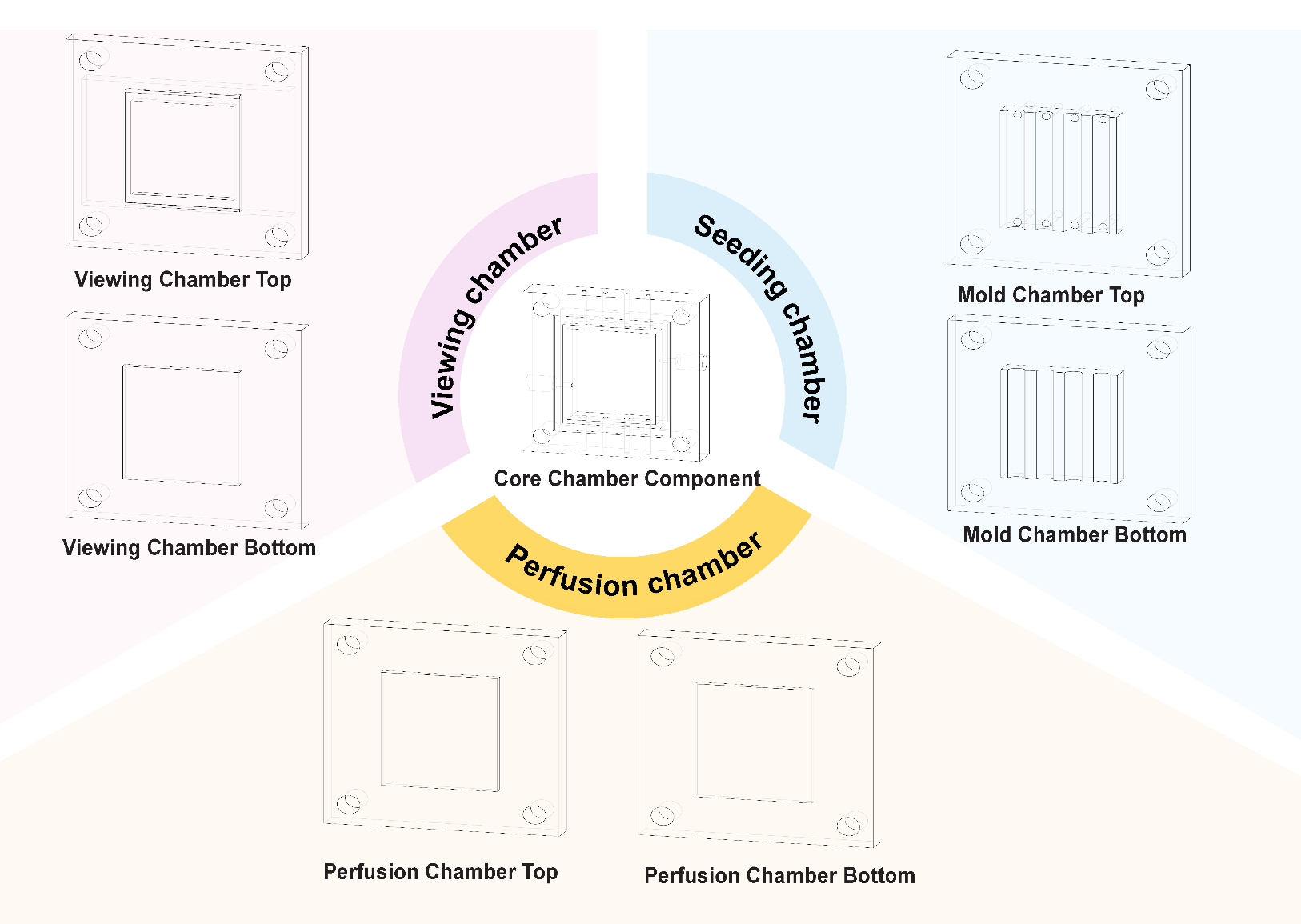


**Fig 1.** **Design and schematic of the TEBV chambers.** The mold, perfusion and viewing chamber are assembled by combining various top and bottom pieces with the core chamber component.

**Procedure**

**Procedure 0: PDMS clamps fabrication and sterilization of TEBV chamber components**

**Timing: 2-3 days before the TEBV fabrication**

**PDMS clamp mold fabrication**

Molds for the PDMS clamps were printed using a Stratsys J750 3D printer with the proprietary VeroPure White filament acrylic formulation. Clean molds thoroughly with sodium hydroxide prior to casting with PDMS.

**PDMS clamps fabrication**

**CRITICAL STEP** Clean the master mold by removing excess PDMS to ensure a flat fit on the dish surface.

1. Prepare SYLGARD 184 silicone Elastomer (PDMS) mixture by adding 5 parts of the elastomer base and 1 part of the curing agent into a 50 mL falcon tube. Inverting the tube several times to ensure thorough mixing of the two components.
2. Centrifuge the tube (1400 rpm, 5 minutes) and degas the mixture for 1.5 hours.

**CRITICAL** No visible bubbles should be present in the PDMS solution.

1. Slowly pour the PDMS into the dish containing the master mold of TEBV clamps. Ensure there is at least 5 mm of PDMS above the top of the molds.
2. Place PDMS-coated molds under vacuum for at least 2 hours to ensure thorough degasification.
3. After degassing, remove molds from vacuum. Using forceps, gently press down so that each is flat against the bottom of the petri dish.
4. Place PDMS-coated molds in an oven set at 50℃ and let them incubate overnight.

**CRITICAL** Ensure the oven temperature does not exceed 55℃ to avoid reaching the glass transition temperature of the VeroPure White molds.

1. Once cured, retrieve the molds from the oven and collectively cut them out of the PDMS using a scalpel.
2. Separate each mold with a straight edge razor, eliminating excess PDMS to leave only 1-2 mm on each side of the molds.
3. With fine-tip forceps, delicately detach the PDMS from all four sides of each mold, taking care to avoid splitting or breaking any part of the PDMS.
4. After separating PDMS from all sides, slowly lift one end of the mold out of the PDMS encasement using fine-tip forceps.

**CRITICAL** Proceed with caution to maintain fidelity of the clamp grooves and prevent PDMS breakage or splitting.

1. Upon complete removal of the mold, trim away the excess PDMS layer from the top of the clamp.
2. Use a straight edge razor to longitudinally cut the clamps on each side, exposing the grooves. Exercise care by positioning the razor appropriately and pressing straight down, ensuring the clamps are approximately 5 mm in width and avoiding excessive cuts on each side.
3. Utilizing a straight edge razor, trim the lateral sides of the clamp, maintaining a gap of approximately 1 mm between the edge of each groove and the clamp's edge.
4. Rotate the clamp 90 degrees, and employing the same technique used for removing excess PDMS from the longitudinal sides, reduce the height of the clamp to 2.75-3 mm.
5. The clamps are now ready for steam sterilization.

**Fabrication of PDMS side port caps**

1. Prepare SYLGARD 184 silicone Elastomer (PDMS) mixture by adding 5 parts of the elastomer base and 1 part of the curing agent into a 50 mL falcon tube. Inverting the tube several times to ensure thorough mixing of the two components.
2. Centrifuge the tube (1400 rpm, 5 minutes) and slowly pour the PDMS into a petri dish.
3. Degas the dish with PDMS mixture for 1.5-2 hours.

**CRITICAL** No visible bubbles should be present in the PDMS solution.

1. Preheat the oven to 50℃ and let PDMS mixture incubate overnight.
2. After curing, carefully remove the dish from the oven. Use a blade to cut out a piece of PDMS at least 0.5 cm in depth.
3. Employ a 2 mm biopsy punch to create a space for covering side ports in the PDMS material.
4. The side port caps are now ready for steam sterilization.

**TEBV mold chamber assembly and materials preparation**

1. Wrap Teflon tape around the two side ports and install them into the side holes on each side of the core chamber component.
2. Use epoxy to attach the eight 0.8’’ stainless steel tubes to the core chamber component and allow the epoxy to cure at room temperature. **CRITICAL** Ensure that the distance between the two ends of the tube on the opposing sides is approximately 1.2 cm, as this determines the length of the TEBVs.
3. Fit the silicone O-rings into the rectangular groove on each side of the core chamber component. **CRITICAL STEP** Ensure that the O-rings are sealed into the grooves on either side of the core chamber component. Replace O-rings and epoxy adhesive after 3-4 uses or if they appear worn.
4. Assemble the top and bottom pieces of the TEBV mold chamber to the assembled core chamber component and then insert four TEBV mandrels into the chamber. **CRITICAL** Make sure that the mandrels do not have any sharp areas that may tear the TEBVs.
5. Sterilize all the following materials prior to use (**Table 1**). All the materials are placed into autoclave pouches unless otherwise indicated.

**Table 1:** Materials list for sterilization.

| **Procedure 1** | Assembled TEBV mold chamber (mold chamber top and bottom, core chamber component, four screws and nuts, four TEBV mandrels, and two PDMS side port caps) |
| --- | --- |
| **Procedure 2** | Viewing/ Perfusion chamber top and bottom; four PDMS clamps; small tubes with varying lengths to replace dysfunctional TEBVs; one pair forceps; stainless steel surgical tray; small square Kimwipes for dehydration; 1 mL syringe with a 23G needle; 5 mL syringe connected to tubing through a luer adapter; Kimwipes for cleanup |
| **Procedure 3** | 1 mL syringe with a 23G needle; one pair forceps |
| **Procedure 4** | Vessel loop tubing; side loop tubing; media reservoir; two pairs of forceps; 5 mL syringe connected to tubing through a luer adapter; Kimwipes for cleanup |
| **Procedure 5** | Two pairs of forceps; 5 mL syringe connected to tubing through a luer adapter; Kimwipes for cleanup |

**NOTE: All the following procedures should be performed in Class II Biosafety cabinets unless specified.**

**Procedure 1: Harvest hNDFs and prepare collagen mixture, Day 0**

**Timing 1 h**

1. Prepare a collagen solution with a final concentration of 7 mg/mL. To achieve this, calculate the required volumes of collagen, 10× DMEM, 1M NaOH, and hNDF media needed for dilution.

The formula for 1 mL collagen and cell mixture for the four TEBV system is

Volume of 10× DMEM =94 μL

Volume of collagen solution (Vc) =1mL× (7 mg/mL/ collagen stock concentration)

Volume 1 M NaOH (V_NaOH_) =Volume of collagen solution × 0.023

Volume of hDNF suspension= 60 μL

Volume of hNDF media (V_hNDF_) =1mL- 94 μL – Vc – V_NaOH_ – 60 μL (if negative then do not add this extra media)

**CRITICAL** May need to aliquot extra volume (>1 mL) to compensate for pipetting error and possible bubble formation.

1. Aliquot the calculated volumes of collagen, 10X DMEM, 1M NaOH solutions in separate sterile 2.5 mL Eppendorf tubes and keep on ice.

**CRITICAL STEP** Avoid making bubbles while pipetting collagen solution.

1. Tighten the nuts on the pre-assembled TEBV mold chamber using pliers. Place on autoclaved surgical tray.

**CRITICAL STEP** Adjust the tightness of the screws in a diagonal, alternating fashion and avoid touching the mandrels. Uneven tightening will cause deformation of the O-ring.

1. Add the calculated volumes of collagen, 10 ×DMEM, 1M NaOH and hNDF media into a sterile 2.5-mL Eppendorf tube and mix gently.

**CRITICAL STEP** Avoid making bubbles while pipetting.

1. Harvest hNDFs by trypsinization and centrifugation. Treat hNDFs (~80% confluent) with trypsin-EDTA for 3 minutes, then add 3 mL fresh DMEM media to neutralize typsin-EDTA. Centrifuge the cells (180 *x g*, 7 minutes). Resuspend the cell pellet in a density of 1×10^6^ hNDFs/60 μL DMEM media.
2. Quickly add 60 μL of hNDF suspension and mix thoroughly by gently swirling the cells in the gel mixture. Then add extra volume of 1M NaOH to adjust pH until the mixture turns magenta (as demonstrated in the video provided in S3).

**CRITICAL STEP** Use a benchtop mini centrifuge to briefly centrifuge the collagen mixture before adding into the mold chamber. Act quickly to avoid collagen gelation.

1. Use 200 μL pipette to slowly add the collagen mixture into each of the 4 vessels of the mold chamber. Stop injecting when the collagen mixture begins to extrude from the contralateral opening.

**CRITICAL** Using a P200 pipette, load 200 μL of the collagen solution, even though each vessel only requires approximately 180 μL. Loading slightly more than the vessel mold capacity ensures that there is enough material to fully fill the mold and compensate for any material lost due to potential bubble formation during injection. Ensure there are no significant bubbles in the mold.

1. Put the mold in a petri dish and seal with parafilm. Incubate in the cell culture incubator for 45-60 minutes.

**? TROUBLESHOOTING**

**Procedure 2: Plastic compression and dehydration, Day 0**

**Timing: ~45 minutes**

1. Remove petri dish containing TEBV mold chamber from incubator to biosafety hood. Remove the chamber from the petri dish. Use pliers to unscrew the nuts and peel off the top and bottom pieces of the mold chamber gently.

**CRITICAL** Avoid touching mandrels on each side during the manipulation to prevent damage to the lumens and contamination.

1. Use forceps to gently apply 2 cm x 2 cm pre-cut sterile Kimwipes directly onto the TEBVs. The Kimwipes should become soaked through as part of the dehydration process. Apply double-layer Kimwipes on each side (~3 times per side) followed by single-layer Kimwipes (~1-2 times per side). Stop the dehydration process once the water absorption on the wipe is isolated to individual TEBVs.

**CRITICAL STEP** Avoid pressing directly on TEBVs during dehydration. Use forceps to gently press the space between TEBVs to ensure uniform fluid dehydration.

**? TROUBLESHOOTING**

1. Assemble the bottom piece of the TEBV viewing chamber and hand tighten the screws. Add 2-2.5 mL warm media into the chamber using a P1000 pipette to soak the dehydrated TEBVs for at least 1 minute. Carefully remove the media from chamber using a P1000.

**CRITICAL STEP** Put pipette tip at the corner of the chamber and slightly tilt the chamber to pull-out media. Avoid touching TEBVs!

1. Firmly secure TEBVs to the mandrels with PDMS clamps using forceps.
2. Hand tighten the top layer of the perfusion chamber first and use plier to gradually tighten the four conners evenly. Tighten the screws in a diagonally alternating pattern.

**? TROUBLESHOOTING**

1. Prime a sterile 5 mL syringe with luer adaptor with warm hNDF media. Invert the syringe to ensure that the media coming out from the syringe is bubble-free. Fill the chamber with media trough the barbed side port using the primed syringe. Tilt the chamber while injecting the media to expel as many bubbles as possible. Cap the side ports using PDMS caps.

**CRITICAL** Use forceps to connect the tube on syringe to the side port to avoid contamination.

1. Remove all the inserted 23RW mandrels carefully using forceps.

**CRITICAL** Pull out the mandrel horizontally, slowing down at both ends to avoid ripping the TEBV lumens during mandrel removal.

1. Perform a leakage test for all the vessels. **CRITICAL** Use a 1mL syringe with a BD syringe adaptor filled with PBS for easy observation. Be attentive to bubbles traversing the TEBV and examine for PBS emerging from the opposite side. If leakage is observed, replace the TEBV with a short section of tubing (see step 10 for details).

**? TROUBLESHOOTING**

1. **OPTIONAL** Place the mold into a petri dish and seal it using parafilm. Inspect each vessel under a phase contrast microscope (5X objective). Look for clear demarcation of the lumen and an intact collagen layer. Replace any vessels showing signs of rupture or a collapsed lumen, as outlined in the step 10.
2. TEBV replacement: Place the chamber on sterile absorbent wipes. Choose the suitable length of sterilized Tygon tubing prior to opening the chamber. Use pliers to open the perfusion chamber. Gently detach the dysfunctional vessel from the connected mandrels using tweezers, ensuring careful handling to prevent displacement of PDMS clamps. Use tweezers to affix the tubing. Seal the perfusion chamber and replenish the chamber with media through the barbed side port. **CRITICAL** Execute the replacement procedure quickly to prevent adjacent vessel damage.

**Procedure 3: Endothelial cell seeding and rotation, Day 0-1**

**Timing: ~ 45 minutes**

1. Prepare a HUVEC suspension of 4.5×10^6^ cells in 600 μL warm EC media. Harvest the cells by using trypsin-EDTA for 3 minutes (~80% confluent), followed by the addition of 3 mL of fresh EC media to neutralize the trypsin-EDTA. Centrifuge the cells at 1000 rpm, 5 minutes.

**CRITICAL STEP** Prepare extra volume of HUVEC suspension for each chamber to compensate for the dead volume of the syringe (~100 μL).

1. Fill a sterile 1-mL syringe with HUVEC suspension and tap to avoid any bubbles until a meniscus is formed at the top of the syringe.
2. Attach the syringe to one end of the vessel mandrel using forceps. Gently push the HUVEC suspension through, perfusing each vessel with ~100 μL of the HUVEC suspension (seeding density: 1.5×10^5^ cell/cm^2^). The cloudy HUVEC suspension drops emerging from the opposite side of the mandrel indicate the presence of HUVECs.
3. Repeat the above step for all the vessels.

**CRITICAL STEP** Tap the syringe for before each injection to ensure that the HUVEC suspension is well mixed.

1. Place the chamber in a dish and seal it with parafilm. Securely affix the chamber to the rotator using lab tape, ensuring that the vessels are parallel to the rotator pole.
2. Set the timer to 12 hours and keep the chamber in the incubator overnight.

**? TROUBLESHOOTING**

**Procedure 4: Perfusion set up, Day 1**

**Timing: ~ 3.5 hours**

There are two perfusion loops for the TEBV chamber as shown in the Fig 2: the vessel loop and the side loop. All the numbers in the parentheses refer to the components in the Fig 2. **CRITICAL** All tubing should be sterilized before TEBV perfusion.

Side Loop Tubing Assembly:

Prepare two pieces of L/S Precision pump tubing with desired length to connect to the MasterFlex pump head. Attach 3-stop microbore tubing to each end of the tubing using a two-way straight connector. Connect the prepared pump tubing to the two-way straight connector (Fig 2, part 1-2-3).

Vessel Loop Tubing Assembly:

Prepare eight pieces of L/S Precision pump tubing and four pieces of Tygon tubing with desired length. Connect a 3-stop microbore tubing with a two-way straight connector on each end of the tubing (Fig 2, part 11-12). Connect one end of the two-way straight connector with a L/S Precision pump tubing (Fig 2, part 10-11-12). Assemble branched tubing by connecting each branch of the Y-shaped connecter with L/S Precision pump tubing (Fig 2, part 10-13). Insert a portion of the small metal mandrel into each Tygon tubing and connect the end branch of the L/S Precision pump tubing, repeating this step four times (Fig 2, part 14-15). Connect branched tubing to the other two-way straight connector. For the outlet tubing of the vessel loop, replace the 3-stop microbore tubing with a L/S Precision pump tubing.

Side Perfusion Loop Setup:

1. Connect one end of the side tubing to the chamber side port first, then prime side loop tubing with warm EC media. **CRITICAL** Clamp the other end of tube securely with forceps to avoid bubbles.
2. Use forceps to connect the side loop tubing to the side port.

Vessel Perfusion Loop Setup:

1. First, prime the outlet tubing by filling it with media and then connect it to the perfusion chamber.
2. Prepare the inlet tubing by filling it with media and secure it by clamping it at the Y-shaped tubing. Any small bubbles trapped in the tubing can be eliminated by either pulsating the syringe plunger or gently squeezing the tubing.
3. Connect each branch of the inlet tubing to the mandrels one by one using forceps.
4. Use forceps to insert the other end of the inlet tubing into the media reservoir.
5. Attach a 0.22 μm syringe filter to the male luer connector on the cap of media reservoir.
6. Set up the pump and start the perfusion at the flow rate of 70 μL/min per TEBV (the readout pump number is 5 rpm). Monitor the media flow from each vessel and replace the vessel if any abnormalities are observed. Adjust the flow rate hourly until it reaches 500 μL/min per TEBV (the readout pump number is 40 rpm).

**CRITICAL STEP** Make sure the flow direction is from the inlet tubing to the outlet.

**? TROUBLESHOOTING**

1. Calculate the wall shear stress $\tau_{w}$ in the TEBV for a given volumetric flow rate (Q, m^3^/s) using the equation $\tau_{w}=\frac{4\mu Q}{\pi R^{3}}$, where R (m) is average TEBV inner lumen radius and μ (Pa•s) is Newtonian fluid of viscosity for cell culture media at 37℃.

**
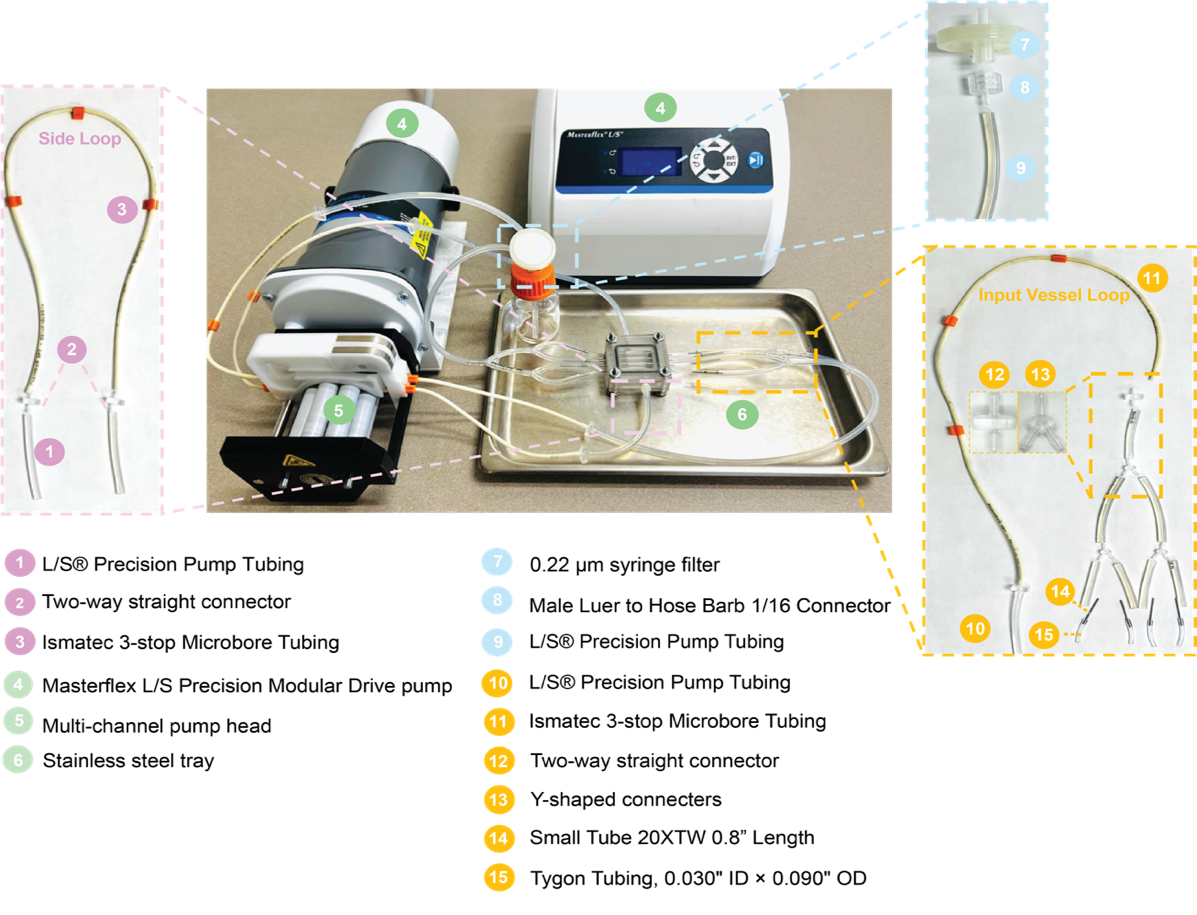
**

**Fig. 2 Perfusion System Configuration.** The tubing and pump arrangement for TEBV perfusion. Ensure all components undergo sterilization and leak testing prior to perfusion. For the output vessel loop, substituting the Ismatec 3-stop Microbore Tubing with L/S® Precision Pump Tubing. Refer to the materials section for detailed product specifications.

**Procedure 5: Daily media change**

**Timing: 5-10 minutes**

1. Disconnect the tubes from the pump and carefully transfer the system into hood.
2. Disconnect one end of the side loop from the barbed side port and place the tube in the waste container.
3. Fill one 5 mL syringe with warm EC media and tap the syringe to remove any bubbles.
4. Use forceps to connect the syringe to the barbed side port and gradually push fresh EC media through the chamber. Meanwhile, expel any bubbles that may form in the chamber during perfusion.
5. Securely clamp the other end of the tube with forceps to prevent the backflow of media. Use forceps to reconnect the tube to the side port.
6. Clamp the inlet tube and aspirate the media in the reservoir.
7. Add 5-10 mL fresh EC media to the reservoir and then remove the clamp.
8. Reconnect the tubes to the pump and monitor the perfusion system to ensure no bubbles are visible in the perfusion loop. **CRITICAL** Make sure to avoid introducing bubbles into the perfusion loops during media changes as this can results in EC detachment and possible TEBV loss.

**Procedure 6: Downstream experiments**

Note the following procedures can be selected and modified according to specific experiment needs. They are not included in the TEBV fabrication and daily maintenance procedures.

**Activation of endothelium and THP-1 monocytes perfusion**

**Timing: 4.5 hours**

1. Change the perfusion media to fresh EC media containing 200U/mL TNF-α, then perfuse the TEBVs at 500 μL/min per TEBV for 4 hours.
2. During the 4-hour endothelium activation step, stain 3×10^6^ THP-1 cells for each chamber of TEBVs using 1μM cell tracker red-CMTPX in warm RPMI media without serum for 30 minutes.
3. **CRITICAL** Following incubation, centrifuge THP-1 cell suspension (1000 rpm, 5 minutes) and wash cells with fresh RPMI media to remove any residual staining reagent.
4. Change the media in the TEBV reservoir before adding stained monocytes. Add THP-1 cells into the main vessel loop at a concentration of 1×10^6^ cells/mL, ensuring a total volume of 3 ml in the media reservoir. **CRITICAL** Avoid adding monocytes in a large volume to prevent cell deposition in the reservoir.

**TEBV fixation**

**Timing: 1.5 hours**

1. Disconnect the chamber from perfusion pump and place the chamber in a chemical fume hood.
2. Load a 5 mL syringe with a luer adaptor attached to a tube with 4% PFA and substitute the medium in the perfusion chamber through the side port. Incubate the vessels for 5 minutes while exchanging the medium in the reservoir with 4% PFA.
3. After the 5-minutes incubation, reconnect the TEBVs to the pump in the chemical fume hood and perfuse vessel lumen with 4% PFA for an additional 5 minutes. Gently open the perfusion chamber and carefully detach the TEBVs from the mandrels using tweezers.
4. Transfer the TEBVs to a 6-well plate and continue fixation (4% PFA) for 1 hour. Rinse the TEBVs three times with PBS and store in PBS at 4 ℃.

**Embedding and sectioning of TEBVs**

**Timing: 2 days**

1. Dehydrate each TEBV in a 5 mL centrifuge tube filled with 30% glucose solution until the vessel sinks at 4 ℃.
2. Remove excess collagen at ends of TEBV then use a 1 mL syringe with a blunt needle to inject a small amount of OCT into the vessel lumen. Stereoscope may be used here to facilitate observation.
3. Transfer the vessels into an embedding cup and hold the vessel vertically using forceps. Gradually fill the cup with OCT.

**CRITICAL** Do not clamp on the vessels while transferring the vessel into the block which leads to closed lumen. Hold the vessel only at the remaining clamped end using tweezers.

1. Carefully position the block flat on dry ice and wait till the OCT turns white.

**CRITICAL** Monitor the vessels and use tweezers to hold them in place as vessels may bend while freezing.

5. Store blocks at -80℃ until ready for cryosectioning.

**Immunofluorescence** **staining of TEBVs**

**Timing: 2 days**

1. For *en face* staining, use a blade to cut a small section of TEBV and delicately insert fine-tip scissors into the vessel lumen to make the incision. Utilize square glass slides during this process to facilitate cutting and opening of the vessels. For TEBV sections, warm the slides to room temperature and rinse off OCT using PBS. Outline the sections using an ImmEdge^TM^ pen.

**CRITICAL** Avoid excessive manipulation during cutting the vessels *en face* as the tools used can damage the integrity of endothelium.

1. For *en face* staining, permeabilize with 0.1% Triton-X in PBS for 30 minutes. For TEBV section staining, permeabilize for 10 minutes. Then rinse samples three times with PBS.
2. Block the TEBV samples with blocking buffer (10% goat serum+10% BSA+ PBS with 0.1% Tween) for 8 hours at room temperature on a shaker. For section slides, block for 1 hour in a humified chamber at room temperature.
3. Add primary antibody at desired dilution ratio in blocking buffer and incubate at 4 ℃ overnight.
4. After primary antibody staining, wash the samples three times with PBS. Subsequently, add secondary antibody at desired dilution ratio in blocking buffer.
5. Samples are rinsed 3 times with PBS before being placed on a glass slide and imaged using fluorescence or confocal microscopy.

**Troubleshooting**

Troubleshooting advice can be found in **Table 2**.

**Table 2:** Troubleshooting table.

| **Step** | **Problem** | **Possible reason** | **Solution** |
| --- | --- | --- | --- |
| Procedure 1  Step 8 | Collagen solution does not gel after 60 minutes incubation. | Improper pH of the collagen solution. | Adjust the pH of the mixture. |
|  | Air pockets form after 60 minutes incubation. | The chamber is not sealed tightly.  Insufficient mixing before injection. | Change the O-ring.  Seal the chamber tightly.  Keep injecting while the collagen solution leaks into the space between two adjacent vessels.  Briefly pipette to mix thoroughly before injection. |
| Procedure 2  Step 2 | Micro bubbles observed in vessel wall after dehydration. | Aggressive pipetting during mixing. | Mix all the components gently in an Eppendorf tube.  Briefly centrifuge before injecting collagen solution into the molds. |
| Procedure 2  Step 5 | Perfusion chamber cannot be tightened evenly. | The PDMS clamps are too thick. | Trim the PDMS clamps.  Change O-ring. |
| Procedure 2  Step 8 | Additional vessels begin to leak following the repair process. | The extraction of media causes a meniscus to form, resulting in increased vessel leakage. | Disassemble the chamber while keeping the media in place and use absorbent Kimwipes to contain the liquid. |
| Procedure 3  Step 6 | Bubbles inside lumen after rotation. | Medium leaks out or evaporates during rotation. | Fix the chamber orientation so that the mandrels are parallel to the rotator platform.  Fabricate PDMS caps to seal the mandrels after the injection of endothelial cell suspension. |
| Procedure 4  Step 8 | Bubbles keep growing in the TEBV perfusion chamber during perfusion. | The chamber does not seal tightly.  Bubbles in the side loop tubing.  Possible leakage in vessels. | Seal the chamber tightly and evenly.  Remove bubbles as much as possible in the chamber and side loop.  Perform leakage test and replace leaky vessels. |

**Timing**

The following timeline assumes fabrication of one chamber of TEBVs. PDMS and tubing materials required for TEBV fabrication and perfusion can be prepared in advance.

**Procedure 0**: PDMS clamps fabrication and sterilize TEBV chamber components 2-3 days

**Procedure 1**: Harvest hNDFs and prepare collagen mixture ~1 hour

**Procedure 2**: Plastic compression and dehydration ~30 minutes

**Procedure 3**: Endothelial cell seeding and set up rotation ~45 minutes

**Procedure 4**: Perfusion set up ~ 3.5 hours

**Procedure 5**: Daily media change 5-10 minutes

**Procedure 6**: Downstream experiments: Activation of endothelium and THP-1 monocytes perfusion

TEBV fixation, *en face* staining of TEBVs

**Note**

Procedures 1 (Harvesting hNDFs and preparing the collagen mixture) and 2 (Plastic compression and dehydration) were modified from the previously published protocol described in reference 4 in the main text.
