# Supplementary material for: Strategies for improved endothelial cell adhesion in microphysiological vascular model systems
Source: PLoS One. 2025 May 19;20(5):e0323080. doi: 10.1371/journal.pone.0323080 (PMC12088046; doi:10.1371/journal.pone.0323080)
Supplement: Attachment — Submitted filename: Response to Reviewers.docx [file pone.0323080.s006.docx]

GEORGE A. TRUSKEY, PROFESSOR

DEPARTMENT OF BIOMEDICAL ENGINEERING	TELEPHONE: (919) 660-5147

1427 FCIEMAS, BOX 90281	FAX: (919) 684-4488

Dear Reviewers,

We are pleased to submit our revised manuscript, “*Strategies for improved endothelial cell adhesion in microphysiological vascular model systems* (**PONE-D-24-55852)**” for consideration for publication in *PLOS One Lab Protocols*. We thank the reviewers for providing helpful feedback and insightful comments. Please find below our point-by-point response to the reviewers. All line numbers referenced correspond to the tracked-changes version of the manuscript.

**Reviewer #1**

1. The rotation speed of the system is described as 15-22 rph and 18-22 rph in different parts of the manuscript. Which is correct and why is there a range?

RESPONSE: We thank the reviewer for the comment. The correct rotation speed range is 18-22 rph. This range reflects the operation of the two custom-made rotators used in our study. Based on our measurements, the average time taken for one rotation is approximately 2.5 minutes, which corresponds to a rotation speed of around 24 rph under ideal conditions. We have included the simulation result for 24 rph in Fig 7C. However, slight fluctuations in speed are expected, and the reported range serves as a practical reference for others who may wish to build similar rotators. We have revised the manuscript to ensure consistency and provide this clarification (lines 115-119).

2. Do the authors expect their optimal conditions to also apply to larger diameter vessels (e.g. 3-6 mm diameter), different endothelial cell types, different biomaterial substrates, different adhesion coatings, and different flow conditions? Might it be necessary to determine optimal seeding conditions for each combination of these parameters?

RESPONSE: While the presented conditions were optimized for the current system, we believe the general conclusions are applicable to other systems. However, due to differences in vessel dimensions, simulations and experiments will be necessary to determine the optimal combinations for varying parameters in other systems. We have included this information in the manuscript (lines 743-745).

3. Given that this is a lab protocol manuscript, the detailed step-by-step protocol provided in the supplement is appreciated. However, this is likely too much detail for the typical reader. I suggest providing a high-level overview of the protocol in the Methods section of the manuscript so readers have sufficient information to understand the rest of the manuscript without needing to read the lengthy supplement.

RESPONSE: We have included a protocol overview in the Methods section (lines 146-161) to provide readers with sufficient information without requiring review of the supplementary protocol.

4. The viewing chamber described in Expected Results section A is difficult to understand based on a text description. Please provide a schematic figure with dimensions and labels. What is the distance between the TEBV lumen and the microscope objective? What is the “frontal housing lens?” Example images using a light microscope would be informative.

RESPONSE: We have added a new schematic figure (Fig 2A) with dimensions and labels to help understand the text description (Fig 2A caption, line 207-214).

5. Please provide CAD files for the chamber, chamber holder, and mold designs in the supplement. This will allow others to fabricate similar devices.

RESPONSE: All CAD files for the chamber, chamber holder, and mold designs are now included in the supplementary materials (S1 CAD files).

6. In Expected Results section B, it would help to clarify the clamping force is provided by snugly assembling the chamber.

RESPONSE: The TEBV chamber should be tightly closed by hand-tightening the four screws located at the corners of the chamber. Then, use pliers to further tighten the screws evenly until the nuts can no longer be turned. We have clarified that the clamping force is achieved by snugly assembling the chamber components (lines 250-251).

7. Please also express the cell seeding densities as cells per cm2 of seeding area. Given the cells are settling onto a surface, the number per area is more useful than the number per volume.

RESPONSE: In this study, the inner radius of the TEBVs was 0.04 cm, resulting in an estimated inner luminal surface area of 0.25 cm² and a lumen volume of 0.005 cm³ for a 1 cm-long TEBV (lines 526-527). Based on these calculations, we have now reported the EC seeding densities as 1.0×10^5^ (low), 1.5×10^5^ (medium), and 2.0×10^5^ (high) ECs/cm² were utilized (lines 287-288). These cell seeding densities have been incorporated into the revised manuscript.

8. Why was en face imaging not performed to calculate cell coverage within the vessels? Why was sectioning performed and circumferential coverage calculated when the chambers nicely provide the ability to directly visualize the cell coverage on the surface of the vessels (e.g. Figure 8B)?

RESPONSE: En face sectioning often leads to endothelial cell loss during processing due to the small size of the vessels. Additionally, the opaqueness of the collagen layer in our viewing chamber prevents accurate evaluation of endothelial coverage *in situ*. Therefore, sectioning and circumferential coverage calculations were deemed the most efficient and reliable methods to evaluate endothelial coverage across the vessel length.

9. Why is there no static group for the data presented in Figure 5C-D?

RESPONSE: In Figure 5, we examined EC coverage after 24 hours of perfusion (Fig 5B) and 7 days of perfusion (Fig 5D). Since the static experiments did not undergo any perfusion, the static attachment conditions alone are not a suitable reference group and were therefore removed. The study in Fig 5C-D emphasizes long-term EC coverage under flow. Due to the oxygen and nutrient supply provided by flowing media, static culture of TEBVs for 7 days would result in substantial cell loss and thus was not included in this figure. Furthermore, dynamic perfusion more closely mimics the physiological conditions of blood vessels, aligning with the setup required for downstream applications.

10. Line 346 may have a typo: starting position in Figure 7A appears to be 90 deg.

RESPONSE: We have corrected the typo.

11. It is stated that rotational speeds above 29 rph prevent cells from contacting the vessel wall. Can these data be shown? E.g. 30 rph?

RESPONSE: We have included an additional figure (Fig 7D) to show cell trajectory under a rotational speed of 30 rph.

12. The last section in Expected Results section C describing the forces acting on the cells reads as disconnected from the rest of the manuscript. Please relate this section back to optimal seedings conditions, especially rotational speed.

RESPONSE: We have revised the manuscript to better relate this section to optimal seeding conditions, particularly rotational speed (line 574-576).

13. Please define the white arrows in the caption for Figure 10A.

RESPONSE: In the revised caption for Figure 10A (line 652), we have clarified that the white arrows indicate adhered monocytes on the endothelial cell surface within the TEBV lumen. This has been clarified in the figure caption.

14. In Figure 1A, consider showing hours/minutes rather than days on the timeline.

RESPONSE: We have modified the timeline in the Figure 1A.

15. In Figure 7, consider highlighting the inner wall located at 0.04 cm.

RESPONSE: We have modified Figure 7 to highlight the inner wall location at 0.04 cm.

**Reviewer #2**:

This study offers an efficient protocol in tissue-engineered blood vessel modeling through the ability to monitor multiple TEBVs simultaneously, developing a viewing chamber for real-time visualization during endothelialization process, simulating the physiological conditions by gradually ramping shear stress, leading to a more stable endothelial layer, demonstrating an optimized process for seeding density and rotation duration, addressing previous gaps in EC adhesion studies and incorporating functional markers (e.g., VE-cadherin, ICAM-1) to confirm endothelial stability and functionality.
All these provide an accessible workflow for vascular research.
So, I recommend this paper for publication in PLOS One journal.

RESPONSE: We are grateful for the reviewer’s recommendation for publication and their acknowledgment of the contributions our protocol offers to vascular research.

In conclusion, we hope these changes adequately respond to the reviewers’ concerns and make the manuscript significantly better. Thank you for your consideration. We look forward to hearing from you.

Yours sincerely,

George A. Truskey, PhD

R. Eugene and Susie E. Goodson

Professor of Biomedical Engineering
