# Supplementary material for: Strategies for improved endothelial cell adhesion in microphysiological vascular model systems
Source: PLoS One. 2025 May 19;20(5):e0323080. doi: 10.1371/journal.pone.0323080 (PMC12088046; doi:10.1371/journal.pone.0323080)
Supplement: Attachment — Submitted filename: Response_to_reviewers_auresp_2.docx [file pone.0323080.s007.docx]

We thank the reviewer for providing additional feedback. Please find below our point-by-point response to the reviewers. The line numbers referenced here correspond to those in the clean manuscript.

1. It is still not clear why the average rotation speed is stated to be 24 rph and the range is stated to be 18-22 rph. How can it be the average speed is not within the range of speeds?

We appreciate the opportunity to further clarify this point. Based on our prior work and the theoretical evaluation of rotational speed during endothelial cell seeding, the initial target range for the rotator system was ~18–22 revolutions per hour (rph). The original rotator, prior to modification, operated at ~ 8 revolutions per minute (rpm).

Upon consultation with our machinist, we determined that the motor supplied with the original rotator was not sufficiently powerful to support the necessary gearing changes to achieve the target speed. Reaching the desired 18–22 rph would have required both replacing the motor and manufacturing a custom worm gear set.

As an alternative, a standard 20:1 worm gear set was available, which reduced the speed to approximately 24 rph. After discussions with our machinist and other team members, we elected to proceed with this readily available option and retain the original motor. The system was then assembled using a custom housing integrated with the rotating assembly. We have included both values in the manuscript to provide practical references for others who may wish to build a similar system and clarified the reasoning behind the discrepancy (line 113-120). To ensure consistency, we addressed this issue in other relevant sections as needed (line 247-248, line 362, line 423-424).

2. It is stated the raw data would be provided in a supplementary file entitled "Raw Data." However, there is no reference to this file in the text of the manuscript and it is not provided as a supplementary file.

Thank you for bringing this issue to our attention. We have included the raw data as the supplementary file. We have also added statement in the figure legends (line 284-285, line 336, line 456-457, line 481-482) to guide readers to the supplementary file entitled "Raw Data."

In conclusion, we appreciate the reviewer’s attention to these issues and hope our explanation resolves the confusion.
